# Cardiovascular Disease and Cardiac Imaging in Inflammatory Arthritis

**DOI:** 10.3390/life13040909

**Published:** 2023-03-30

**Authors:** Anastasia-Vasiliki Madenidou, Sophie Mavrogeni, Elena Nikiphorou

**Affiliations:** 1 Centre for Musculoskeletal Research, School of Biological Sciences, Faculty of Biology, Medicine & Health, The University of Manchester, Manchester M13 9PL, UK; 2 Cardiology Clinic, Onassis Cardiac Surgery Centre, 176 74 Athens, Greece; 3 University Research Institute of Maternal and Child Health and Precision Medicine, Medical School, National and Kapodistrian University of Athens, Aghia Sophia Children’s Hospital, 115 27 Athens, Greece; 4 Center for Adolescent Medicine and UNESCO Chair in Adolescent Health Care, Medical School, National and Kapodistrian University of Athens, Aghia Sophia Children’s Hospital, 115 27 Athens, Greece; 5 Centre for Rheumatic Diseases, School of Immunology and Microbial Sciences, King’s College London, London WC2R 2LS, UK; 6 Rheumatology Department, King’s College Hospital, London SE5 9RS, UK

**Keywords:** rheumatoid arthritis, cardiovascular disease, psoriatic arthritis, axial spondylarthritis, imaging, inflammation

## Abstract

Cardiovascular morbidity and mortality are more prevalent in inflammatory arthritis (IA) compared to the general population. Recognizing the importance of addressing this issue, the European League Against Rheumatism (EULAR) published guidelines on cardiovascular disease (CVD) risk management in IA in 2016, with plans to update going forward based on the latest emerging evidence. Herein we review the latest evidence on cardiovascular disease in IA, taking a focus on rheumatoid arthritis, psoriatic arthritis, and axial spondylarthritis, reflecting on the scale of the problem and imaging modalities to identify disease. Evidence demonstrates that both traditional CVD factors and inflammation contribute to the higher CVD burden. Whereas CVD has decreased with the newer anti-rheumatic treatments currently available, CVD continues to remain an important comorbidity in IA patients calling for prompt screening and management of CVD and related risk factors. Non-invasive cardiovascular imaging has been attracting much attention in view of the possibility of detecting cardiovascular lesions in IA accurately and promptly, even at the pre-clinical stage. We reflect on imaging modalities to screen for CVD in IA and on the important role of rheumatologists and cardiologists working closely together.

## 1. Introduction

Mortality rates in patients with inflammatory arthritis (IA), such as rheumatoid arthritis (RA), radiographic spondyloarthritis (r-axSpA), and psoriatic arthritis (PsA), are higher than in the general population [1,2,3,4,5]. Cardiovascular death has been recognized as a leading cause of mortality in IA, accounting for up to 40% of all deaths [2,4,6]. Cardiovascular morbidity is only partially explained by traditional cardiovascular risk factors, with inflammation driving the cardiovascular disease (CVD) risk [7,8,9]. Inflammatory mediators, such as interleukin-18 in PsA and IL-6 in RA, have been proven to contribute to the CVD burden [10,11]. 

Newer medications that are effective in suppressing inflammation, such as Tumor Necrosis Factor inhibitors (TNFi), have been associated with lower CVD risk [12,13,14]. However, although cardiovascular mortality may have decreased with the better management of inflammatory arthritis, cardiovascular mortality is still high, necessitating aggressive CVD risk management [1,3,15,16,17]. In 2009, a European Alliance of Associations for Rheumatology (EULAR) task force formulated 10 recommendations for the screening and management of CVD in inflammatory arthritis [18]. In view of emerging new evidence, the CVD risk management recommendations were updated in 2016 [19]. In this narrative review, we assess the current state of cardiovascular disease in inflammatory arthritis.

## 2. Materials and Methods

We searched PubMed for English papers published from inception to 27 December 2022, using the MeSH terms “rheumatoid arthritis”, “psoriatic arthritis”, “spondylarthritis”, “ankylosing spondylitis”, and “cardiovascular disease”, yielding 10,921 reports. From the retrieved manuscripts, 172 articles were included, comprising randomized controlled trials, observational (excluding case reports), and basic science studies prioritized by study quality and relevance, systematic reviews, and meta-analyses. We particularly focused on papers that have been influential in informing the landscape of cardiovascular disease in inflammatory arthritis and the role of cardiovascular imaging. 

## 3. Results

### 3.1. Rheumatoid Arthritis

Cardiovascular disease risk in patients with RA is substantially elevated compared with the general population [20,21]. The risk of CVD in RA is comparable to diabetes mellitus (DM), a well-recognized risk factor of CVD with established guidelines about the management of CVD risk [22,23,24]. 

Cardiovascular disease in RA is associated with cardiovascular death. According to a systematic review from 2009, RA patients have increased CV mortality compared with age- and the sex-matched general population [standardized mortality ratio (SMR) 1.61 (95% CI 1.48–1.75)] [25]. A later study from the Netherlands investigating the CV mortality of RA patients between 1997 and 2012 showed similar results [26]. The authors found a higher risk of CV mortality at 24% compared to the general Dutch population [2]. Although the SMR decreased by 3% annually, the decrease was not statistically significant (*p* = 0.16) [26]. 

On the other hand, a study based on the Oslo RA register found that CVD mortality was not significantly increased over the 10-year follow-up in RA patients diagnosed after 2004 [16]. The results from Oslo may indicate that newer treatments have a positive effect on mortality in patients with RA. The role of anti-rheumatic treatments on CVD is better illustrated in a Finish study which assessed the CV mortality in early RA patients diagnosed between 2000 and 2007 with a reported CV mortality SMR of 0.57 (95% CI 0.52–0.62) [27]. The use of glucocorticoids was associated with a higher risk of CV death, whereas the use of methotrexate with a lower risk [27].

#### 3.1.1. Cardiovascular Risk Factors

The occurrence of new CV events in RA is at least partially explained by traditional CVD factors [28,29,30]. A systematic review from 2015 found that hypertension, diabetes mellitus, smoking, hypercholesterolemia, and obesity increased the CV risk in patients with RA [31].

More specifically, a large cohort of early RA patients found that hypertension was the strongest risk factor for cardiovascular events [32]. An Italian study with 21,201 RA patients identified that hypertension was more common in RA than in non-RA subjects (*p* < 0.001) [29]. In 2021, a systematic review of fourteen studies found that methotrexate and exercise are associated with reduced risk of incident hypertension in RA patients [33]. On the other hand, prednisolone and (cyclooxygenase-2) COX-2 inhibitors seem to increase the risk of incident hypertension in RA patients [33].

Metabolic syndrome (MetS) is a constellation of metabolic abnormalities that includes central obesity, glucose intolerance, insulin resistance, high blood pressure, and dyslipidemia [34]. A systematic review published in 2022 estimated the pooled prevalence of MetS in RA at 32% (95% CI 29.6–34.4) [33]. In other words, one in three RA patients has MetS. MetS was more common in studies conducted in Asia (32.7%, 95% CI 29–36.3) and Europe (32.7%, 95% CI 27.5–37.9) and less prevalent in studies related to Africa (28%, 95% CI 28.8–32.2) [35]. Marinque-Arija et al. from Spain found that the presence of insulin resistance in patients with RA was associated with obesity [Odds Ratio (OR) 6.01, 95% CI 1.9–8.7] and higher cumulative disease activity score during follow-up (OR 2.8, 95% CI 1.3–6.0) [36]. 

UK data show the prevalence of obesity to be increased from 13.3% in 1990 to 33.6% in 2010 [37]. In Canada, a multicenter study of early RA patients found that the minority of the population had normal Body Mass Index (BMI), with 35% being overweight and 33% obese [38]. Overweight and obese patients were 25% and 47% less likely, respectively, to achieve sustained remission in the first 3 years, despite similar treatments [38]. The negative association between obesity and the likelihood of achieving low disease activity was observed in a large UK study with two multicenter RA inception cohorts [39]. A population study from Denmark showed that for women, the overall RA risk was 10% higher for each 5% increment of total body fat (HR 1.10, 95% CI 1.02–1.18), 5% higher for each 5 cm increment of waist circumference (HR 1.05, 95% CI 1.01–1.10), and nearly 50% higher in those who were obese compared to normal BMI (HR 1.46 95% CI 1.12–1.90) [40].

Focusing now on diabetes, a UK population study showed that the incidence rate for diabetes was 6.3 cases per 1000 person-years among individuals with RA [41]. An Italian study with 21,201 RA patients found that diabetes was more common in RA than in non-RA subjects (10.2 vs. 9.6%, *p* = 0.004) [29]. A multi-national study found that atherosclerotic CVD was present in 26.7% of RA patients with DM compared with 11.6% of patients without DM (*p* < 0.001) [42]. It also seems that CVD risk factors cluster. A higher number of RA patients with DM had hypertension and hyperlipidemia and were on lipid-lowering or antihypertensive agents than the non-diabetic RA patients (*p* < 0.001 for all) [42]. An Italian study showed that diabetes was associated with a higher risk of subclinical (OR 4.50, 95% CI 1.74–11.62) and clinical (OR 6.21, 95% CI 2.19–17.71) atherosclerosis [43]. 

Dyslipidemia is a disorder of lipoprotein metabolism that results in high low-density lipoprotein cholesterol (LDL-C), high triglycerides, and low HDL-C. A study from Spain found that the prevalence of dyslipidemia was 54.9% in RA [44]. Boyer et al. conducted a systematic review of traditional cardiovascular risk factors in RA patients [45]. The authors found that HDL cholesterol levels were lower in RA patients, with a weighted mean difference at −17.72 mg/dL (*p* < 0.00001) [45]. Zhang et al., from the USA, also found that HDL-cholesterol ≥ 1.6 mmol/L compared with <1.0 mmol/L was associated with reduced Myocardial Infarction (MI) risk (HR 0.37, 95% CI 0.21–0.66) in RA patients [46]. On the contrary, the aforementioned meta-analysis found that the prevalence of hypercholesterolemia was not significantly higher in RA [45]. Further, an Italian study found that dyslipidemia was more frequent in the non-RA group than in RA patients (16.5% vs. 15.4%, *p* < 0.001) [29]. 

A meta-analysis with 2956 RA patients on CV risk factors found that smoking is more prevalent in RA patients compared with controls (OR 1.56, 95% CI 1.35–1.80) [45]. A multi-national cohort study in RA showed that current smokers at enrolment were more likely to have moderate or high disease activity (70.6%) compared with former and never smokers (62.5% and 60.4%, respectively, *p* < 0.001 for both) after adjusting for confounders [47]. As expected, former and never smokers had fewer CVD event rates than current smokers (HR 0.70, 95% CI 0.51–0.95, and 0.48, 95% CI 0.34–0.69, respectively) [47]. 

In summary, traditional cardiovascular risk factors seem to confer a significant burden on cardiovascular disease among patients with RA. Of note, traditional CV risk factors, such as obesity, metabolic syndrome, and smoking, seem also to be associated with higher disease activity. Consequently, aggressive assessment and management of conventional cardiovascular risk factors are required for all RA patients, signposting where appropriate patients to other members of the multi-disciplinary team and relevant support groups.

#### 3.1.2. Inflammation and (Subclinical) Cardiovascular Disease

The excess risk of CVD events in RA is the result of the synergy between traditional CVD factors and inflammation. A UK study based on a population of 1,475,762 people found that patients with RA were more likely to have a history of MI, stroke, or heart failure before the diagnosis of RA [48]. The high CVD risk increases further post-diagnosis and is not attributed to traditional CVD risk factors [46]. Lauper et al., from Switzerland, found that the incidence and prevalence of major adverse cardiovascular events in RA were not statistically significantly different from axial spondylarthritis (axSpA) and PsA after adjusting for confounding factors [49]. These findings could suggest that inflammation, rather than a particular type of arthritis, accounts for the increased risk of CVD [49]. 

Carotid intima-media thickness (CIMT) is a non-invasive imaging test used to assess the thickness of the intima and media of the carotid arteries [50]. It has been widely used as a surrogate marker of atherosclerotic disease and is a strong predictor of CV events [50]. The presence of carotid plaques is considered an even more reliable predictor of CV events than CIMT [51]. A systematic review (n = 4317 RA patients) showed that RA patients had a higher common carotid CIMT (mean difference 0.10 mm, 95% CI 0.07–0.12) and an increased prevalence of carotid plaques (OR 3.61, 95% CI 2.65–4.93) compared to the control group [52]. 

The evidence shows that the more severe the inflammatory status, the higher the CVD burden is. Meta-regression models showed that higher disease activity (as expressed by DAS-28, CRP levels, and ESR) was associated with the higher common carotid CIMT in RA patients (*p* = 0.003) [52]. Using the population-based data resources of the Rochester Epidemiology Project, the authors found that the risk of cardiovascular death was significantly higher among RA patients with at least three recorded Erythrocyte Sedimentation Rate (ESR) values of > or =60 mm/h (HR 2.03, 95% CI 1.45–2.83), RA vasculitis (HR 2.41, 95% CI 1.00–5.81), and RA lung disease (HR 2.32, 95% CI 1.11–4.84) after adjusting for cardiovascular risk factors and comorbidities [53]. An early RA cohort from the Netherlands showed that time-averaged DAS-28 was significantly associated with CVD (*p* = 0.002) [54].

The particularly interesting question remains whether the cardiovascular risk decreases with the control of inflammation. A recent systematic review of 19 articles found that TNFi seems to reduce the risk of CV events in RA [55]. The QUEST-RA project found that the risk of CV disease decreases with prolonged use of treatments such as methotrexate, sulfasalazine, leflunomide, glucocorticoids, and TNF inhibitors [56]. It also seems that improvement in disease activity reduces the CV risk based on a longitudinal US-based registry [57]. Although tofacitinib, a JAKi, is an effective treatment for RA, a post-authorization safety trial found a higher risk of major cardiovascular events (MACE) for Tofacitinib patients compared to TNFi [58]. 

### 3.2. Axial Spondyloarthritis (Radiographic and Non-Radiographic Axial Spondyloarthritis)

Patients with axSpA have an increased risk of cardiovascular events and mortality [59,60,61]. A meta-analysis from 2021 found that SpA patients have a significantly higher risk of MI [Risk Ratio (RR) 1.52, 95% CI 1.29–1.80) and stroke (RR 1.21, 95% CI 1.0–1.47) than the general population. However, this higher CV risk did not significantly increase the overall mortality risk (RR 1.23, 95% CI 0.96–1.57) [62]. 

The international Assessment in SpondyloArthritis International Society (ASAS)-COMOSPA study, a multi-national study of SpA patients, evaluated the prevalence of comorbidities in SpA [63]. The study found that the global prevalence of ischemic heart disease and stroke in the study population was substantial, at 2.7% (95% CI 2.2–3.2) and 1.3% (95% CI 0.9–1.7), respectively [63].

#### 3.2.1. Cardiovascular Risk Factors

Traditional cardiovascular risk factors account partially for the increased cardiovascular disease in AxSpA [64]. A cohort study of 6262 r-axSpA patients from Taiwan found that hypertension, diabetes mellitus, and hyperlipidemia are more prevalent in AS patients than in the control group. Further, the overall incidence rate of the acute coronary syndrome (ACS) was higher in the AS patients than in the non-AS cohort (4.4 vs. 2.9 per 1000 person-years), with an adjusted hazard ratio of 1.36 (95% CI 1.16–1.59) [59].

The aforementioned (ASAS)-COMOSPA study found that hypertension was the most prevalent CV disease risk factor in AS patients at 33.5% (95% CI 32.0–35.0), particularly in the Northern European countries [63]. Smoking was the second most prevalent risk factor at 29.3% (95% CI 27.9–30.7) [63]. The prevalence of smoking was similar at 24% in the BSR biologics register for AS (BSRBR-AS, which recruited axSpA patients) [65]. An observational cohort study based on the Danish nationwide DANBIO registry found that current and previous smokers had significantly lower odds of responding to treatment than never smokers [OR 0.48 (95% CI 0.35–0.65) and 0.53 (95% CI 0.35–0.80), respectively] [66]. Dulger et al. from Turkey conducted a mono-centric study where they divided the AS patients into two groups, those who quit smoking (group 1 a = 17) and those who did not (group 1 b = 37). The baseline data and data 6 months after smoking cessation were compared. Although the baseline parameters were similar between the two groups, disease activity in 6 months was significantly better in AS patients who stopped smoking (*p* = 0.02) [67]. Smoking also seems to affect the response to TNF inhibitors negatively [68]. 

A multicenter study from Ireland found that obesity was the most prevalent chronic condition at 37% in a population of 734 axSpA patients [69]. Mass et al., from the Netherlands (n = 461 axSpA patients), found that 37% of the population was overweight and 22% obese. Moreover, obese patients had significantly higher disease activity, worse physical mobility, and worse quality of life than overweight and normal-weight patients [70]. Similar findings were also observed by Chen et al. (association between obesity and higher inflammation, disease activity, and impaired physical function) [70]. Interestingly, Chen et al. identified that central obesity could predict a patient’s longitudinal radiographic change [71].

Glucose intolerance in the form of insulin resistance or diabetes mellitus may contribute to the increased CV risk in AS patients. A study based on the database of the Taiwan National Health Insurance Program found that the frequency of diabetes mellitus was 1.21-fold higher in the r-axSpA group than in the non-radiographic axSpA spondylitis group (1.43 vs. 1.19 per 100 person-years, 95% CI 1.02–1.43, *p* = 0.025) [72]. Kiortsis et al., from Greece, assessed the effects of infliximab infusions on insulin sensitivity in 28 patients with RA and 17 with AS (non-diabetic) after 6 months of treatment [73]. The Homoeostasis Model Assessment (HOMA) Index, a measure of insulin sensitivity, and the Quantitative Insulin Sensitivity Check Index (QUICKI), a measure of insulin sensitivity, were used. The group of patients with the highest insulin resistance had a significant decrease in the HOMA index and an increase in the QUICKI (*p* < 0.01 for both indexes) with no significant differences between the RA and AS patients [73]. Another study from Spain found similar results in terms of QUICKI and HOMA index values after the use of infliximab in non-diabetic AS patients [74]. 

Metabolic syndrome (MetS) may be more prevalent in AS patients compared to the general population. In a study of 63 men with AS, Papadakis et al. found that the prevalence of the MetS was higher in AS patients compared to age-matched controls (34.9% vs. 19.0%; *p* < 0.05) [75]. The prevalence of MetS in AS was slightly lower but higher than the control group in a Brazilian study (27% vs. 9.1%, *p* = 0.04) [76]. An earlier Italian study of 24 AS patients estimated the frequency at 45.8%, significantly higher than the controls (10.5%, *p* = 0.019) [77].

Dyslipidemia seems also to contribute to the CVD risk in AS. The previously described (ASAS)-COMOSPA study found that the global prevalence of hypercholesterolemia was 27.3% (95% CI 25.9 to 28.7) [63]. Malesci et al., from Italy, found that triglyceride to HDL cholesterol ratio (*p* = 0.002) and LDL cholesterol (*p* = 0.03) were significantly higher in AS patients than in controls [77]. In line with the other lipid abnormalities, HDL cholesterol was significantly lower (*p* < 0.001) [77]. Kucuk et al., from Turkey, investigated the relationship between LDL/HDL ratio and carotid intima-media thickness (CIMT), a surrogate marker of atherosclerosis. A higher LDL/HDL ratio was seen in AS patients compared to the control group (2.85 ± 1.00 and 2.47 ± 0.90, respectively, *p* = 0.047) [78]. As expected, a positive correlation was found between carotid intima-media thickness (CIMT) and LDL/HDL [78].

In conclusion, the collective evidence suggests that smoking, hypertension, dyslipidemia, obesity, glucose intolerance, and metabolic syndrome potentially contribute to increased cardiovascular morbidity in axSpA patients. 

#### 3.2.2. Inflammation and (Subclinical) Cardiovascular Disease

Traditional cardiovascular risk factors do not solely contribute to axSpA patients’ increased cardiovascular mortality and morbidity. In keeping with other inflammatory arthritides, atherosclerotic disease is now considered to play a major role in the high cardiovascular morbidity observed in axSpA patients. Inflammation seems to have a pivotal role in atherogenesis, leading to cardiovascular events if it remains untreated [79]. AS and non-radiographic axSpA seem to have similar atherosclerosis burdens [80]. 

High disease activity is associated with CVD. A multicenter study from Spain found that CRP > 3 mg/L and ESR at the time of diagnosis were significantly associated with CIMT, a surrogate marker of atherosclerosis [81]. Another study from Italy showed that the persistence of increased CRP levels and high disease activity (BASDAI > 4 or ASDAS > 2.1) are associated with increased CVD [82]. 

On the other hand, TNF inhibitors may stabilize or slow down the progression of subclinical atherosclerosis and endothelial dysfunction in AS patients [83,84]. As might be expected, a meta-analysis from 2021 found that low disease activity in AS is not associated with accelerated atherosclerosis [85].

#### 3.2.3. Other Cardiovascular Abnormalities and Potential Association with CVD 

Axial spondyloarthritis is associated with cardiac abnormalities outside of ischaemic heart disease, such as aortitis and conduction disturbances. Inflammation is not confined to the musculoskeletal system but can affect the aorta causing aortitis (AR). The frequency of AR is calculated at 2 to 12% in patients with r-axSpA, most of whom had long-lasting disease [86]. Aortitis can cause aneurysms and even aortic root dilation when the ascending aorta is involved leading to aortic valve regurgitation and heart failure [87]. In a study of 65 participants, sacroiliitis has been associated with aortic inflammation and has been shown to be a predictor of CVD regardless of traditional CV risk factors [88]. 

Conduction disturbances (CDs) in r-axSpA are well- described in the literature for over 30 years, although the prevalence has been debated [89,90,91,92]. In terms of pathophysiology, inflammation and/or fibrosis may affect the atrioventricular (AV) node and both bundle branches, causing conduction disturbances [93,94]. Two studies have found that disease duration is associated with cardiac abnormalities, which could suggest that chronic, untreated inflammation in the cardiac tissue causes conduction abnormalities [89,90]. In a cross-sectional study of CDs in 57 patients with rheumatic diseases (not only IA), the authors found that the higher prevalence of CDs in the patient population is associated with cardiovascular disease [95].

Although the evidence is sparse, inflammation outside the skeleton and peripheral joints may contribute to the higher risk of CVD in r-axSpA. 

### 3.3. Psoriatic Arthritis

There is growing evidence that patients with psoriatic arthritis are at higher risk of cardiovascular events [96,97,98]. In a systematic review, Horreau et al. reported a higher risk of myocardial infarction (MI) in PsA patients compared with the general population (OR 1.57, 95% CI 1.08–2.27) [99]. A study from Toronto with 648 patients showed that the standardized prevalence ratio for angina in PsA patients is higher than the Canadian Community Health Survey at 1.97 (95% CI 1.24–3.12) [100]. A systematic review from 2021 showed that PsA patients also have a higher risk of stroke with RR at 1.33 (95% CI 1.22–1.45) [101]. 

Cardiovascular events are a leading cause of death in PsA [96,102]. A recent systematic review from Chaudhary et al. found that cardiovascular mortality is significantly higher for PsA patients compared to the general population (RR 1.21, 95% CI 1.06–1.38) [103].

#### 3.3.1. Cardiovascular Risk Factors

In 2021, a systematic review with a sample size of 150,677 patients found that the most prevalent comorbidities in PsA were hypertension (pooled prevalence 34%), metabolic syndrome (29%), obesity (27%), and hyperlipidemia (24%) [104]. All these comorbidities are actually cardiovascular risk factors that contribute to the higher prevalence of cardiovascular disease in PsA patients.

Hypertension is a CV risk factor more common in PsA patients compared to the general population [105]. In a large Middle-Eastern PsA cohort (n = 3161), hypertension was more prevalent compared to the control group (OR 1.51, 95% CI 1.40–1.62). Data from a Spanish study showed that the prevalence of HTN was higher in PsA compared to healthy controls (OR 1.3, 95% CI 1.0–1.8) [106]. A Canadian study (n = 449) showed that hypertension was more common in PsA compared to psoriasis (OR 2.08, 95% CI 1.02–4.25) [107]. 

A study from the MarketScan database showed that the incidence rate of hyperlipidemia per 1000 patient years in PsA was 40.3 (95% CI 39.4–41.3) [108]. A higher prevalence of dyslipidemia has been observed in PsA compared to the general population [105,109,110,111]. Interestingly, a US study with 95,540 participants showed that participants with hypercholesterolemia lasting for ≥7 years were at a higher risk of developing psoriasis with PsA (HR 1.68, 95% CI 1.12–2.52) and psoriasis (HR 1.29, 95% CI 1.03–1.61) [112]. 

Obesity is another common cardiovascular risk factor in psoriatic disease [113,114,115]. Obesity is a negative predictor of therapeutic response in PsA [116,117], and on the other hand, evidence shows that weight loss improves disease activity [118]. A Canadian study found that PsA patients with higher BMI were less likely to achieve sustained low disease activity compared to those with normal BMI after adjusting for confounders [119]. A US study with 89,049 participants showed that the risk of PsA is higher in overweight and obese patients. Compared with BMI less than 25.0, the PsA risk is getting significantly higher for each BMI category, with the highest one at 6.46 (95% CI 4.11–10.16) for BMI ≥ 35.0 [120]. Overall, it seems that obesity is not only associated with increased CVD but affects the trajectory of the disease.

A systematic review from 2021 found that the pooled prevalence of MetS in PsA populations was 0.46 ± 0.06 (95% CI 0.40–0.51) [121]. PsA Patients were also 1.62 (95% CI 1.50–1.74) and 1.66 (95% CI 1.54–1.79) times more likely to have MetS compared with PsO and RA populations [121]. Further, some studies have shown that insulin resistance in patients with psoriasis is improved after the introduction of TNF inhibitors [122,123,124]. The literature on PsA is sparse [3], and therefore, we cannot draw any robust conclusions.

Type 2 diabetes mellitus is more common in patients with PsA compared to the general population, with the prevalence ranging from 6.1 to 20.2% [125]. The link between PsA and DM is not fully understood, but TNF-a seems to contribute to the development of insulin resistance [126,127]. 

Overall, several conventional cardiovascular risk factors appear to be present in PsA patients. Of note, rheumatologists tend to focus on how other conditions (comorbidities) affect PsA, such as the association between obesity and CVD risk in PsA. However, as described above, insulin resistance may be improved by treatments for psoriatic arthritis, and obesity increases the risk of developing PsA. The above findings on CVD risk factors highlight the interplay of coexisting conditions. As Dey et al. suggested for RA, we may need to move away from the term comorbidity, indicating the presence of an index disease as the center of interest, and focus on the concept of multimorbidity where all diseases are managed equally, and a comprehensive approach to care is considered [128]. 

#### 3.3.2. Inflammation and Cardiovascular Disease

Cardiovascular disease in PsA patients is not solely explained by traditional risk factors, but chronic inflammation is an important contributing factor [129,130]. It seems that active disease suggestive of higher levels of inflammation contributes even more. Juneblad et al., from Sweden, found that Disease Activity Index (DAI) was significantly associated with death from diseases of the circulatory system (OR 1.86, 95% CI 1.20–2.89) [131]. A monocentric Spanish study showed that a high number of swollen joints during the evolution of psoriatic arthritis is associated with CVD compared to non-inflammatory patients (OR 2.9, 95% CI 1.1–8.0) [111]. Data from China showed that high CRP, suggestive of increased levels of inflammation, was significantly associated with CV events (HR 1.02, 95% CI 1.00–1.04) after adjusting for baseline Framingham risk score (FRS) [132]. 

Patients with psoriasis alone also have a higher burden of CVD. In a systematic review, severe psoriasis was associated with a significantly increased risk of cardiovascular mortality (RR 1.39, 95% CI 1.11–1.74), myocardial infarction (RR 1.70, 95% CI 1.32–2.18), and stroke (RR 1.56, 95% CI 1.32–1.84) [133]. A retrospective study from the Clinical Practice Research Datalink database (n = 27,672 psoriasis patients) found that patients with mild psoriasis have fewer comorbidities than those with severe psoriasis, and patients with psoriasis are less affected by comorbidities than those with PsA [134]. Gladman et al. showed that cardiovascular disease in PsA is associated with more severe psoriasis (high Psoriasis Area) [100]. Maybe the additive effect of the inflammation from psoriasis contributes to the overall cardiovascular risk of PsA patients.

The above findings suggest the idea that targeting inflammation should have a favorable effect on cardiovascular risk in patients with PsA. A systematic review from 2016 found that the risk of cardiovascular events was markedly decreased in the TNF inhibitor group compared with MTX treatment in PsA and psoriasis patients (RR 0.67, 95% CI 0.52–0.88) [15]. TNF inhibitors may also improve the endothelial function of PsA patients [135]. 

#### 3.3.3. Subclinical Cardiovascular Disease

As traditional CV risk factors cannot fully account for the increased CVD risk in PsA, it is apparent that assessment of CV risk solely based on traditional CV risk factors is insufficient. Therefore, early and appropriate methods for CV risk assessment in psoriatic arthritis are of paramount importance [136,137]. 

A systematic review from Jamil et al. found a significant association between subclinical atherosclerosis and PsA [138]. Methods such as CIMT, PWV, coronary calcification score (CCS), and ankle-brachial index (ABI) values were used to assess the extent of atherosclerosis. The most commonly used method was the b-mode ultrasound that measures IMT [138]. A study based on the CORRONA registry (n = 4672) found a significant association between disease activity in PsA and pro-atherogenic lipid profile [139]. The role of inflammation in atherosclerosis is better illustrated in an Irish study with 50 PsA patients [140]. Szentpetery et al. found that the highest C-reactive protein level, highest swollen joint count, and disease duration, but not metabolic syndrome, were independent predictors of higher coronary plaque formation in PsA [140]. On the other hand, a Chinese study showed that low disease activity at 12 months had a protective effect on atherosclerotic plaque progression (OR 0.27, 95% CI 0.09–0.84) [141].

### 3.4. Screening for CVD in Inflammatory Arthritis and the Role of the Rheumatologist

Screening for CVD in patients with inflammatory arthritis is imperative, given the robust evidence of increased CVD risk across RA, PsA, and axSpA, as described above. Against this background, the European League Against Rheumatism (EULAR) task force formulated 10 recommendations for the screening and management of CVD in inflammatory arthritis (IA) [19]. Here, we reflect on the EULAR guidelines taking into consideration the European Society of Cardiology (ESC) guidelines and the latest evidence.

First and foremost, the rheumatologist should ensure that CVD risk management is performed in patients with inflammatory arthritis [19]. According to the EULAR guidelines, CVD risk assessment is recommended for all patients with IA once every 5 years or earlier if there is a major change in antirheumatic treatment or they have intermediate CVD risk [19]. This recommendation is in line with the 2016 ESC guidelines [142]. 

The SCORE risk calculator is recommended for CVD risk evaluation in the general population by the ESC guidelines [142]. As this calculator takes only into account the traditional risk factors, the EULAR guidelines suggest the use of a 1.5 risk multiplier to capture the inflammatory burden in IA, which translates into a higher CVD risk [19]. On the other hand, the ESC guidelines are more conservative, and they recommend the use of the 1.5 risk multiplier for autoimmune diseases other than RA may be considered on an individual level, depending on disease activity/severity [142]. 

QRISK2 is another CVD risk algorithm recommended by the British Societies’ consensus recommendations for the prevention of cardiovascular disease (JBS3) [143]. This predictor incorporates a multiplication factor of 1.4 for RA patients [143]. In 2017, an updated version of QRISK2, called QRISK3, was developed and validated [144]. 

The EULAR guidelines recommend that the CVD risk assessment in RA could include screening for asymptomatic atherosclerotic plaques with the use of carotid ultrasound [19]. In 2021, a Spanish study with 990 consecutive RA patients aimed to investigate if the combined application of the QRISK3 and modified SCORE as per EULAR guidelines are better at identifying carotid plaques, a surrogate marker of atherosclerosis, than each one [145]. Based on their positive findings, the authors proposed the combined use of both risk predictors in RA [145].

Lifestyle measures, such as a healthy diet, regular exercise, and smoking cessation, are recommended for all IA patients by the EULAR guidelines [19]. More studies supportive of lifestyle documentation have been published since the development of the EULAR guidelines. In 2020, Roeelsgaard et al. found that smoking cessation in patients with RA was a predictor of reduced rates of CVD events [47]. In 2021, Hulander et al. investigated if an anti-inflammatory diet is cardioprotective in weight-stable patients with RA [146]. The results demonstrated significant improvement in blood lipid profile (triglycerides, HDL) and Apo-B100/Apo-A1 ratio, which are important markers of future CVD risk [146,147]. A randomized controlled study of 112 RA patients found that dietary education was associated with significant improvements in multiple traditional CVD risk factors (cholesterol, triglycerides, LDL, systolic and diastolic blood pressure) [148]. 

With regards to rheumatology management, the latest EULAR guidelines advocate that the prescription of NSAIDs in RA and PsA should be with caution, especially for patients with known CVD or CVD risk factors [19]. This recommendation was mainly based on a systematic review of multiple antirheumatic medications and cardiovascular events in RA and PsA [13]. Roubille et al. found that NSAIDs increased the risk of all CVEs (RR 1.18, 95% CI 1.01–1.38), but this may have been specifically related to the use of rofecoxib (withdrawn from the market in 2004) [13]. 

The aforementioned systematic review also found that corticosteroids increased the risk of all CVEs (RR 1.47, 95% CI 1.34–1.60) in PsA and RA patients [13]. Therefore, it is not surprising that the EULAR guidelines suggest that the lowest effective dose of glucocorticoids be prescribed for prolonged periods and glucocorticoids be decreased in case of minimal disease activity [19].

Finally, EULAR guidelines recommend that disease activity should be controlled optimally in IA patients to reduce CVD risk [17]. This is in line with the accumulating evidence that inflammation is associated with CVD risk in IA patients, as described in the previous sections. 

## 4. Role of Non-Invasive Imaging Modalities in the Evaluation of Cardiovascular Disease in Inflammatory Arthritides

**Figure 1:**
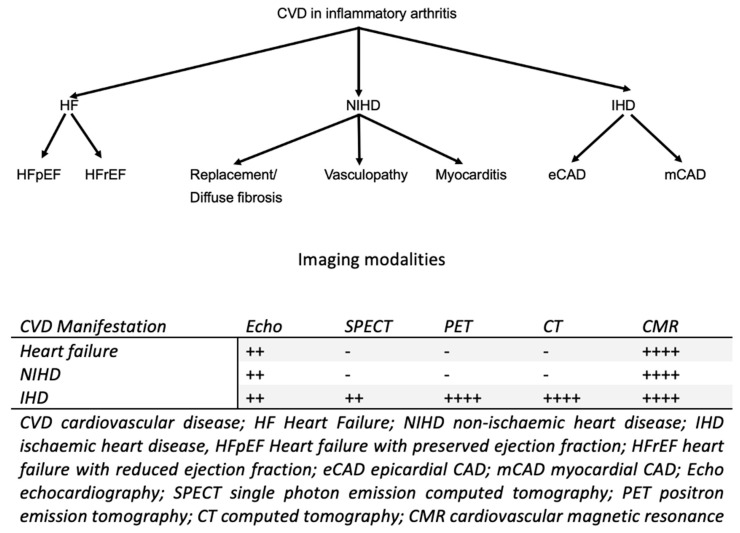
Shows the main cardiac pathophysiologic phenomena taking place during the course of inflammatory arthritis and the best-performing imaging modalities in each clinical scenario. ++: good imaging modality for each CVD manifestation. ++++: very good imaging modality for each CVD manifestation.

In the sections that follow, we focus on the currently used cardiovascular imaging modalities for the assessment of various aspects of CVD in IA (Figure 1). The main clinical parameters that should be evaluated to establish the diagnosis of CVD include.

### 4.1. Biventricular Function Assessment

The functional status of both ventricles is essential to establish CVD involvement in IA. In this context, echocardiography is the routinely used imaging modality to assess biventricular function in clinical cardiology because it is widely available, low cost, used at the bedside, and with great acceptance among cardiologists. However, it is an operator, and acoustic window depended on modality, which means that it has low reproducibility, depending on the operator’s experience and also the patient’s body size [146]. Furthermore, it is of limited value in the evaluation of right ventricular status, which is of great importance in IA [149]. In contrast to echocardiography, cardiovascular magnetic resonance (CMR) is the ideal modality to assess biventricular function independently of body size and operator experience [149]. This is even more important when we design research studies because, in CMR, the sample size needed using CMR is significantly lower compared to echocardiography, which leads to better cost-effectiveness [149].

### 4.2. Wall Motion Evaluation

This approach can be used in both subclinical and clinical CVD in IA. Speckle tracking echocardiography (STE) has been successfully used for the assessment of subclinical LV dysfunction, specifically in patients with RA [150]. CMR images can also be analyzed using feature-tracking techniques. However, the results of the two modalities are not interchangeable, and each modality has its own limitations [151].

### 4.3. Myocardial Ischemia Assessment

Myocardial ischemia may be due either to epicardial coronary artery disease (e-CAD) or microvascular coronary artery disease (m-CAD). Stress echocardiography (SE) is a useful tool with similar diagnostic and prognostic value in comparison to radionuclide stress imaging. However, it is more cost-effective and does not need the use of ionizing radiation. Coronary flow reserve (CFR), assessed by Doppler echocardiography, can diagnose the presence of micro-macro-vascular disease in patients with RA [152,153], although it has not been routinely used. 

In contrast to CFR, exercise and pharmacological SE are the two main methods used in clinical practice to assess for myocardial ischemia. Dobutamine is the most commonly used agent for pharmacological SE [154]. It increases heart rate 2- to 3-fold, systolic BP 2-fold, and contractility 4-fold through activation of β-1 receptors. The positive inotropic effect of dobutamine makes this stress agent the preferred pharmacologic stress for the evaluation of contractility reserve [155]. SE is an established method for the evaluation of symptomatic/asymptomatic patients with valvular heart disease, CAD, and cardiomyopathies providing important information about risk stratification [155]. However, we should always remember that echocardiography has the limitation of the acoustic window and operator dependency. Furthermore, patients with severe arrhythmia cannot be evaluated using this test. 

In parallel with SE, nuclear techniques can also be used for the evaluation of myocardial ischemia. Between them are included Single-photon emission computed tomography (SPECT) and Positron Emission Tomography (PET). SPECT was used to assess RA patients with suspected CAD, but a meta-analysis showed that Cardiovascular Magnetic Resonance (CMR) had better diagnostic value for the detection of CAD [156]. The wide availability of SPECT in Europe/the USA and the strong prognostic data supporting its use in the evaluation of e-CAD make this modality a valuable tool for ischemia evaluation [156].

However, SPECT has certain limitations, including exposure to ionizing radiation, the presence of imaging artefacts, and the low spatial resolution, which do not allow for the quantification of subendocardial ischemia and small scars [149]. Recently, the MR-IMPACT trial and the CE-MARC study support the wider use of CMR for the assessment of CAD rather than SPECT due to the higher diagnostic accuracy of CMR and the concerns about ionizing radiation, particularly in young women who need serial follow-up [157]. In a meta-analysis comparing myocardial perfusion assessment with SPECT, PET, and CMR, all modalities presented a high sensitivity (88–91% on a patient basis) but with significant differences in specificity [158]. SPECT had the lowest specificity, while PET and CMR achieved the highest diagnostic performance, with stress CMR providing an important alternative without ionizing radiation [159]. In the current European and American guidelines, all non-invasive modalities are recommended at similar levels [160].

PET and PET/computed tomography (PET/CT) can also be used for ischemia evaluation. PET/CT has been routinely used in oncology but only recently in cardiology. It is known that FDG is concentrated in tumor cells. However, macrophages also demonstrate increased FDG uptake, which is indicative of vascular inflammation. Therefore, ascending aortic FDG uptake has been identified in RA patients without clinically overt CVD [161]. In patients with IA, reduced coronary vasodilator reserve, identified by PET, was associated with worse CVD outcomes and all-cause mortality [162]. Furthermore, PET/CT revealed inflammatory and metabolic factors to subclinical CAD in PsA, including adipose inflammation suggesting novel targets for CVD prevention and treatment in SpA [163]. However, until now, its routine use in clinical practice is limited by the lack of availability, high costs, the use of ionizing radiation, and the lack of expertise [149].

CMR can also play an important role in ischemia detection. CMR diagnostic value is based on the difference of contrast perfusion in myocardium perfused by obstructed vs. unobstructed coronary arteries. It is known that coronary artery stenosis does not allow the increase of myocardial perfusion during hyperemia, provoked by the administration of the vasodilator factor adenosine. This is imaged as a perfusion defect during stress, which is not visible at rest, compared with the myocardium, perfused by unobstructed coronary arteries. In the presence of fibrosis, the perfusion defect, identified during stress, remains stable during rest [163]. Compared to PET, quantitative perfusion CMR has the advantage of wide availability, better spatial resolution, lower cost, and lack of ionizing radiation [163].

### 4.4. Non-Invasive Coronary Artery Evaluation

Computed tomographic angiography (CTA) is currently routinely used for the non-invasive evaluation of epicardial coronary arteries and large vessels. Compared with age-matched controls, patients with RA had a greater prevalence of asymptomatic CAD, with higher coronary calcium score (CCS), more frequent multivessel disease, as well as more high-risk plaques [164]. Furthermore, the use of biologic disease-modifying anti-rheumatic drugs (bDMARDs) has been associated with reduced CVD risk based on CTA findings [165]. Lastly, the application of FDG-PET/CT to estimate the presence of vascular inflammation supports epidemiologic data identifying premature atherosclerosis in psoriasis (PSO) and RA [166,167]. However, high costs, use of ionizing radiation, and use of nephrotoxic contrast agents are important limitations of CT to be considered [149].

### 4.5. Myocardial Edema-Fibrosis Assessment

Myocardial edema can be assessed and quantified only by CMR [149]. Since myocardial inflammation is of great clinical significance in IA [149], the capability of CMR to differentiate acute from chronic myocardial involvement has a great impact on the diagnostic and therapeutic approach. Furthermore, myocardial fibrosis is of great value for the CVD evaluation of IA patients because it is part of inflammatory, ischemic, or nonischemic myocardial disease and may be expressed either as a replacement or as diffuse fibrosis. Echocardiography can provide an indirect assessment of fibrosis based on wall motion findings (hypo-or akinesia). Nuclear techniques can also provide information about fibrosis. However, the low spatial resolution, the inability to perform tissue characterization, and the need for ionizing radiation make these techniques suboptimal for the accurate evaluation of fibrosis in IA.

**Figure 2:**
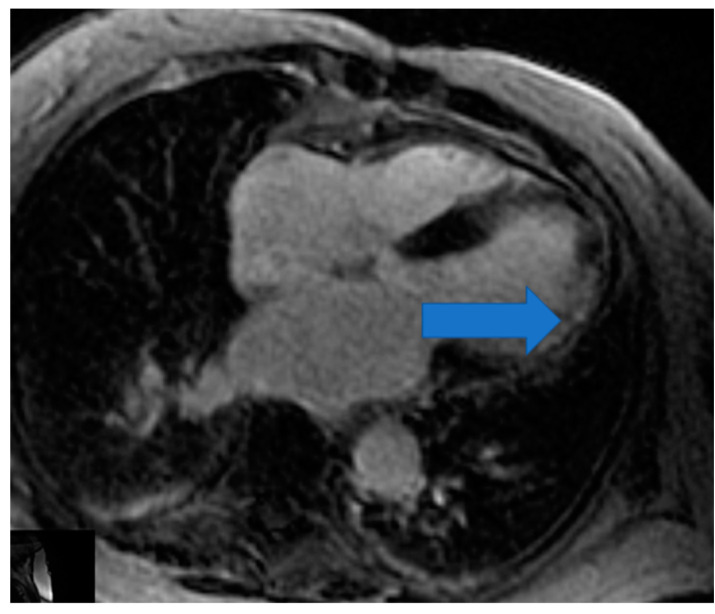
Cardiovascular Magnetic Resonance (CMR) showing subendocardial myocardial infarction in the lateral wall of LV (Left Ventricle) in RA (Rheumatoid Arthritis). Image was obtained with permission from the Olympic Diagnostic Research Centre.

**Figure 3:**
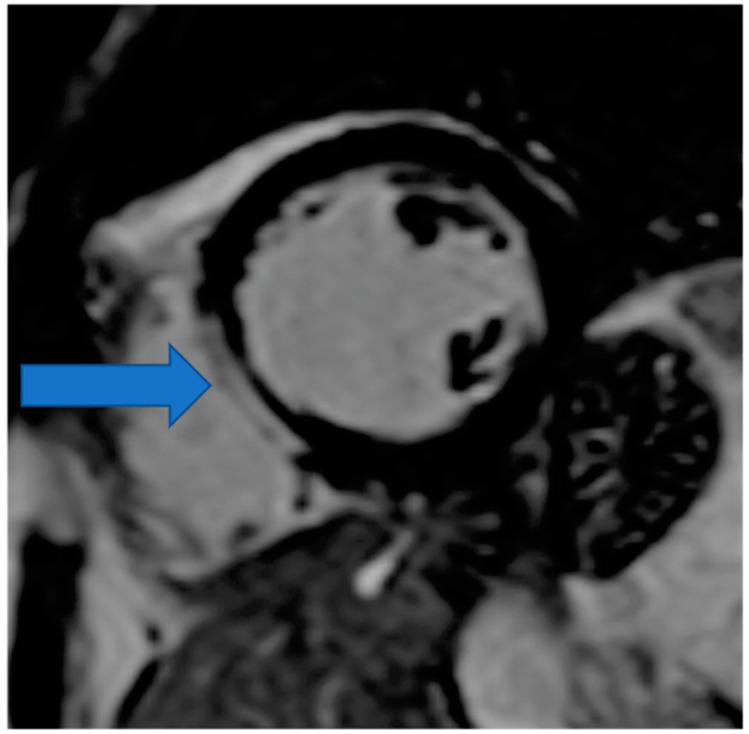
Cardiovascular Magnetic Resonance (CMR) showing myocardial inflammation and subepicardial LGE (late gadolinium enhancement) in the interventricular septum in RA (Rheumatoid Arthritis). Image was obtained with permission from the Olympic Diagnostic Research Centre.

**Figure 4:**
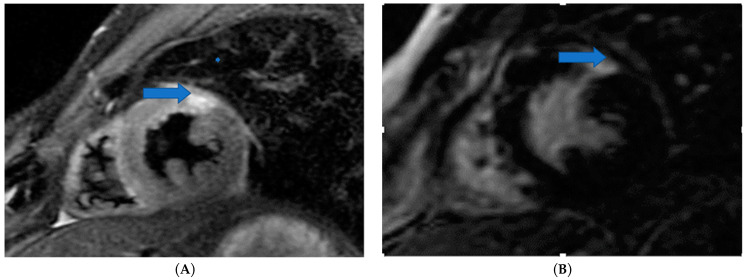
Cardiovascular Magnetic Resonance (CMR) showing acute myocardial infarction and normal coronary arteries MINOCA) in systemic sclerosis: (**A**) STIR2 image for edema detection (**B**) Inversion recovery image showing LGE (late gadolinium enhancement) in the same area due to myocardial infarction. Image was obtained with permission from the Olympic Diagnostic Research Centre.

**Figure 5:**
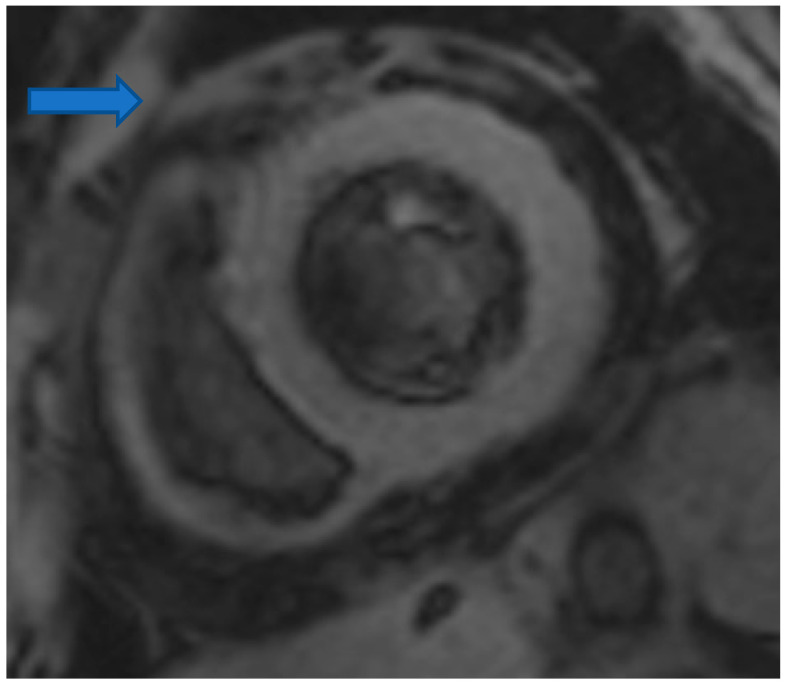
Cardiovascular Magnetic Resonance (CMR) showing diffuse myocardial edema and pericardial effusion in systemic sclerosis (STIRT2 image). All the LV myocardium is thick, and therefore it was initially considered hypertrophy. However, the STIRT2 image clarified that it was edema with a myocardial over skeletal muscle ratio equal to 2.8 (normal values < 2). Image was obtained with permission from the Olympic Diagnostic Research Centre.

**Figure 6:**
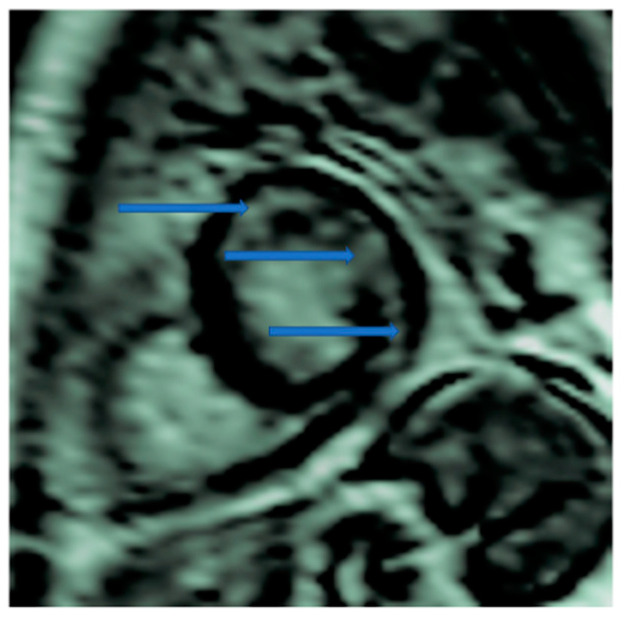
Cardiovascular Magnetic Resonance (CMR) showing extensive intramyocardial LGE (late gadolinium enhancement) due to replacement fibrosis in systemic sclerosis (inversion recovery image). The patient presented with ventricular tachycardia. Image was obtained with permission from the Olympic Diagnostic Research Centre.

CMR is the only non-invasive imaging modality capable of providing fibrosis information equal to that provided by myocardial biopsy [149]. In this regard, CMR can assess replacement fibrosis using late gadolinium T1-enhanced imaging (LGE) (subendocardial, due to CAD in Figure 2, and subepicardial, due to inflammation in Figure 3, respectively). However, IA may also present diffuse myocardial fibrosis with or without concurrent LGE. Diffuse fibrosis can be detected using native T1 mapping and extracellular volume fraction (ECV) quantification. These indices have a good correlation with the results of the myocardial biopsy, and native T1 mapping can also be used as a non-contrast index of fibrosis in those patients with contraindications to paramagnetic contrast agents [149]. Currently, there are no evidence-based data regarding the role of various imaging modalities in the prevention, diagnosis, and treatment assessment of ARDs. However, a consensus-based decision algorithm for when a CMR study could be considered in patients with autoimmune rheumatic diseases (ARDs), together with a standardized study protocol, has recently been published [149]. Representative CMR images from various clinical scenarios are included in Figure 2, Figure 3, Figure 4, Figure 5 and Figure 6.

### 4.6. Future Directions

New promising cardiac imaging approaches are described below.

1. **Oxygen-sensitive CMR imaging**. This technique has been correlated with endothelial function and inducible myocardial ischemia and has recently been suggested as a means of assessing myocardial vascular function and improving the diagnosis of vasculopathy in cardiac allografts [168]. The inflammatory mechanisms seen with ARDs may impair endothelial function, as shown in cardiac allografts, and oxygen-sensitive imaging may offer an interesting and novel approach to studying these diseases.
2. **Molecular imaging**. The use of superparamagnetic iron oxide (SPIO) nanoparticles for cellular tracking has been attracting much interest [169]. Briefly, when imaging immune-mediated diseases such as multiple sclerosis, leukocytes, most often macrophages, are labeled with SPIO through either in vivo or in vitro phagocytosis or with transfection agents under MR imaging, a blooming artefact can be seen. This effect is mediated through T2 signal reduction; in one study, the magnitude of T2 signal reduction was found to correlate with macrophage content in the atherosclerotic plaque. As the abundance of leukocytes in cardiac tissue can indicate the severity of the condition, cell-labeling approaches with SPIO have potential useful implications for ARDs [169].
3. **Hyperpolarized magnetic resonance (MR).** This is an emerging imaging technology that generates contrast agents with 10- to 20,000-fold improvements in MR signal, enabling cardiac metabolite mapping. The use of hyperpolarized MR using [1–^13^C] pyruvate provides a novel method for the assessment of innate immune cell-driven inflammation in the heart after myocardial infarction, with broad potential applicability across other cardiovascular and inflammatory diseases and suitability for early clinical translation [167]. Furthermore, an experimental model of re-perfused myocardial infarction could be of value for testing new drugs to treat reperfusion injury and facilitate translational research [170].
4. **Cardiac energetics using Magnetic Resonance Spectroscopy (MRS).** Magnetic resonance spectroscopy (MRS) is a non-invasive tool to analyze cardiac energy metabolism both in clinical and pre-clinical settings.

This unique tool can be utilized to assess metabolic alterations in a wide range of cardiovascular diseases, including myocardial ischemia/inflammation/fibrosis [168]. However, it is not without limitations, and better and faster techniques are needed to map relevant metabolites such as phosphate [171,172].

## 5. Conclusions

To conclude, IA is associated with a higher prevalence of CVD due to both traditional CV risk factors and inflammation. Although the use of new anti-rheumatic treatments has been immensely helpful in better-controlling inflammation, the latter is not always possible in all patients, maintaining the high risk of CVD. Table 1 summarizes key findings based on the existing evidence base. Aggressive screening for CVD and management of CV risk factors is crucial to reduce cardiovascular morbidity and mortality in IA. Non-invasive cardiovascular imaging and vascular assessment, such as CIMT, offer great potential to detect early and accurately the preclinical cardiovascular lesions in IA. The choice of each modality should be individualized according to the clinical scenario, the patient’s clinical status as well as the experience of the team with various imaging modalities. Currently, CMR is the only non-invasive, radiation-free modality that can offer the possibility of “non-invasive tissue biopsy”, providing direct information about ischemia, edema, and fibrosis, independently of blood biomarkers. Rheumatologists and cardiologists should work closely together to optimize the care of patients with IA and cardiovascular comorbidity, addressing the latter even at the pre-clinical stage. 

## Figures and Tables

**Table 1:** Key messages.

1. Cardiovascular disease (CVD) risk is increased in inflammatory arthritis (rheumatoid arthritis, psoriatic arthritis, axial spondyloarthritis) compared to the general population.
2. CVD is associated with increased cardiovascular mortality and morbidity.
3. CVD results from a combination of traditional CV risk factors and inflammation.
4. Traditional CV factors, such as obesity, diabetes, and dyslipidemia, seem to confer a significant burden on cardiovascular disease among patients with inflammatory arthritis (IA).
5. Aggressive screening for CVD, using available tools, such as the SCORE risk calculator, is recommended.
6. Management of CV risk factors is crucial to reduce cardiovascular morbidity and mortality in IA.
7. Disease activity should be optimally controlled, given the pivotal role of inflammation in CVD.
8. Non-invasive cardiovascular imaging modalities offer great potential to detect preclinical lesions in IA.
9. Collaboration between rheumatologists and cardiologists is important for more comprehensive care in patients with IA and cardiovascular disease.

## Data Availability

All data are included in the manuscript.
